# Contact impedances of textile electrodes for electrical impedance tomography

**DOI:** 10.1186/2197-425X-3-S1-A274

**Published:** 2015-10-01

**Authors:** AD Waldmann, KH Wodack, A März, A Ukere, C Trepte, SH Böhm, DA Reuter

**Affiliations:** Swisstom AG, Landquart, Switzerland; University Medical Centre, Hamburg-Eppendorf (UKE), Hamburg, Germany

## Introduction

In thoracic electrical impedance tomography (EIT) electrodes are placed around the human thorax. Weak alternating currents are applied via two electrodes and the resulting potentials are sensed by the remaining electrodes. The measured voltages are then converted into moving real-time images showing heart and lung functions rather than its structures. EIT is non-invasive and can thus be employed over several hours right at the bedside. However, for mid and long term measurements stable electrical electrode skin contact and a skin friendly patient interface are required.

## Methods

In this study the electrical skin contact of a novel textile-based electrode assembly comprising 32 active electrodes integrated in a vest-like structure was assessed in 10 healthy volunteers and in 40 patients undergoing robot-assisted radical prostatectomy. The study protocol was approved by the ethics committee of the medical board Hamburg (PV374) and written consent was obtained from all patients. The electrode assembly was connected to the Swisstom BB^2^ and the skin contact impedance was measured once with dry and once with wetted electrodes in the 10 healthy volunteers. An electrically non-conductive oil-in-water liquid emulsion, called ContactAgent (CA), was used to wet out the textile electrodes. Mid-term stability was then measured over a period of 4 hours in the 40 patients. To avoid any undue burden measurements with dry electrodes were not repeated in patients.

## Results

Skin contact impedance of the dry electrodes was higher than 2'000 Ohm. After applying the contact agent, skin contact impedance dropped below 500 Ohm and remained there for the next 4 hours.

## Discussion and Conclusions

In spite of being non-conductive, the CA markedly decreased skin contact impedance. Possible explanation are that the CA increases the adherence of the textile electrode to the skin, increases the effective electrode contact area while displacing the air pockets both, within the yarns of the textile layer and between the electrode and the skin thereby improving the dielectric properties of the skin-electrode system. We conclude that the new patient interface in combination with the special CA provides a stable electrical contact between electrodes and the skin for at least 4 hours.

**Figure 1:**
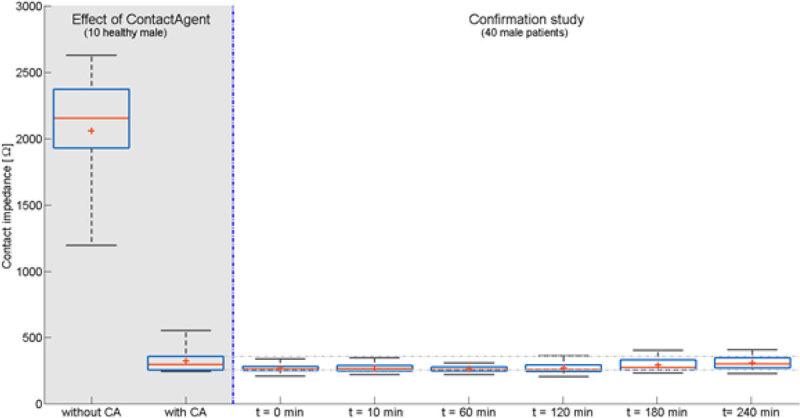
**Skin contact impedance box plot using a six point description: minimum (black line), first quartile (blue line), mean (red cross), median (red line), third quartile (blue line), and maximum (black line).** The left part of the plot - grey shaded area - shows the effect of the CA in 10 healthy volunteers. The median skin contact impedance values decreases from 2'151 Ohm in dry textile electrodes to 299 Ohm after applying the CA. The mean values decreases from 2'056 ± 464 Ohm to 325 ± 88 Ohm. Right part of the plot depicts the contact impedances measured in patients. During the 4 hours observation period in 40 male patients contact impedance increases slightly.

